# Use of Combined MSAP and NGS Techniques to Identify Differentially Methylated Regions in Somaclones: A Case Study of Two Stable Somatic Wheat Mutants

**DOI:** 10.1371/journal.pone.0165749

**Published:** 2016-10-28

**Authors:** Miroslav Baránek, Jana Čechová, Tamas Kovacs, Aleš Eichmeier, Shunli Wang, Jana Raddová, Tomáš Nečas, Xingguo Ye

**Affiliations:** 1 Mendeleum–Department of Genetics, Horticulture Faculty of Mendel University in Brno, Lednice, Czech Republic; 2 Enviroinvest Co., Pecs, Hungary; 3 Institute of Crop Sciences, Chinese Academy of Agricultural Sciences, Beijing, China; 4 Department of Fruit Growing, Horticulture Faculty of Mendel University in Brno, Lednice, Czech Republic; Nanjing Agricultural University, CHINA

## Abstract

The appearance of somaclonal variability induced by *in vitro* cultivation is relatively frequent and can, in some cases, provide a valuable source of new genetic variation for crop improvement. The cause of this phenomenon remains unknown; however, there are a number of reports suggesting that epigenetics, including DNA methylations, are an important factor. In addition to the non-heritable DNA methylation changes caused by transient and reversible stress-responsive gene regulation, recent evidence supports the existence of mitotically and meiotically inherited changes. The induction of phenotypes via stable DNA methylation changes has occasionally great economical value; however, very little is known about the genetic or molecular basis of these phenotypes. We used a novel approach consisting of a standard MSAP analysis followed by deep amplicon sequencing to better understand this phenomenon. Our models included two wheat genotypes, and their somaclones induced using *in vitro* cultivation with a changed heritable phenotype (shortened stem height and silenced high molecular weight glutenin). Using this novel procedure, we obtained information on the dissimilarity of DNA methylation landscapes between the standard cultivar and its respective somaclones, and we extracted the sequences and genome regions that were differentially methylated between subjects. Transposable elements were identified as the most likely factor for producing changes in somaclone properties. In summary, the novel approach of combining MSAP and NGS is relatively easy and widely applicable, which is a rather unique feature compared with the currently available techniques in the epigenetics field.

## Introduction

Epigenetics is a relatively new scientific field that is currently undergoing a meteoric rise. Breakthrough articles dealing with aspects of epigenetics in cancer therapy, human diet and plant sciences have been published. Chromatin remodeling and direct DNA methylation or demethylation processes act as epigenetic regulators. Both of these pathways are likely directed by small RNAs [1]. To better understand the epigenetic influence on various phenomena, methods for assessing DNA methylation and chromatin modification have been developed. Histone modifications are studied by using chromatin immunoprecipitation (ChIP) of associated DNA, followed by amplification of cDNA using polymerase chain reaction (PCR) or whole genome microarray hybridization [2,3,4]. However, alterations in DNA methylation are the main measure used to determine the influence of epigenetic factors [5,6].

To study global DNA methylation, which is defined as the ratio of methylated deoxycytosines to total deoxycytosines, high performance liquid chromatography (HPLC) or high performance capillary electrophoresis (HPCE) is typically employed [7]. Methods based on bisulfite conversion are used to investigate sequence-specific DNA methylation because treating DNA with bisulfite converts unmethylated cytosines into uracil while leaving methylated cytosines unchanged [8,9]. Another option for DNA methylome analysis is a strategy using methylated DNA immunoprecipitation followed by high-throughput sequencing (MeDIP-seq) [10]. With the rapid rise in popularity of techniques known as NGS (Next Generation Sequencing) recently emerged publications, where NGS principle is used also to study genome changes due to tissue culture plant regeneration. In principle they work as extensions to previously described methods such as bisulfite sequencing (mantled genotype) or they are entirely new approaches. For example, heritable genomic variations as SNPs, small scale insertions/deletions (Indels) and mobilization of transposable elements (TEs) being induced by tissue culture in rice were studied by using whole genome re-sequencing [11]. Another a high-throughput sequencing technology, known as Single Molecule Real Time Sequencing (SMRT), was established as a tool to directly determine the methylation status of a analyzed DNA strain [12,13]. However until now there is no reference about SMRT application for plant methylome studies.

A different principle is utilized for the methylation-sensitive amplified polymorphism (MSAP) protocol. The MSAP technique uses a pair of methylation-sensitive restriction enzymes that recognize the same sequence but have differential sensitivity to methylation at cytosines within the recognized site. Selective PCR amplification and subsequent comparison of the spectra generated by each enzyme of the isoschizomer pair allows the determination of the cytosine methylation status at the restriction sites, which are typically CCGG loci. Despite some unfavorable properties of the MSAP method, this technique continues to be reliable epigenetic tool used in high-quality studies [14,15,16]. As a nonspecific DNA-based marker system, the MSAP method can provide information on the similarity of DNA methylation states between subjects but not the sequence of the individual amplicons. The other approach, known as metAFLP is also based on AFLP technique [17]. It overcomes limitations of the MSAP by giving numerous characteristics affecting restriction sites including sequence changes and site DNA methylation variation (demethylation and de novo methylation) in a single experiment. The approach was used in numerous studies including i.e. triticale [18] or *Gentiana pannonica* [19]. In both cases, however, to determine the characteristics of amplicons with polymorphisms, it is necessary to perform a subsequent sequencing of polymorphic products [20,21,22]. Nevertheless, this is experimentally very demanding and time consuming and can increase the risk for errors (e.g., cutting of bands composed from more amplicons or unsuccessful sequencing of the amplicon of interest).

Somaclonal variation in plants cultivated under *in vitro* culture conditions is significantly influenced by epigenetic factors, including DNA methylation changes; however, the purpose of these modulatory effects are unclear [15,20,23]. The occurrence of somaclonal variability is relatively frequent and, in some cases, provides a valuable source of genetic variation for crop improvement. For example, newly selected variants may exhibit resistance to biotic or abiotic factors that can lead to improved quality or higher yield [24,25,26,27]. On the other hand there are references that indicate limited stability of the plant materials derived via tissue cultures if they pass through several generative cycles [28]. Thus, when planning activities based on inducing variability by in vitro, it is needed to take that risk into account. Regarding our current study, it is important to note that shortened stems and dwarfism in different plant species were repeatedly induced under *in vitro* conditions [29,30,31,32]. In the case of wheat, this dwarfing phenotype is an extremely important qualitative parameter that significantly increases breeding value. For example, the discovery of two major semi-dwarfing genes in the common wheat cultivar Norin 10 in the 1960s revolutionized wheat production by dramatically improving lodging resistance and grain yield [33].

Based on previous studies, it is increasingly evident that induced changes in DNA methylation should be divided into two classes based on their stability. Non-heritable changes are caused by transient and reversible stress-responsive gene regulation and allow somaclones to acclimate to stressful *in vitro* conditions. But DNA methylation changes can also be mitotically or meiotically inherited [34,35]. Though stable DNA methylation changes can induce economically valuable phenotypes, little is known about the genetic or molecular basis of these changes. Similarly, information about the ratio of stable/heritable epigenetic modifications to reversible DNA modifications is quite scarce. Baránek et al. [20] found that 40% of the changes seen immediately after *in vitro* cultivation disappeared after one year of planting in flowerpots. The remaining 60% of DNA methylation changes persisted within these one year old regenerants; thus, this figure likely represents mitotically inherited events. In the current study, two wheat genotypes with somaclones that showed a) a shortened stem height and b) silenced high molecular weight glutenin subunits (HMW-GSs) were analysed. Both of these somaclone types were repeatedly self-pollinated to confirm the stability and heritability of the two induced phenotypes [36,37]. These somaclones are ideal study materials to elucidate the genomic regions that are repeatedly changed by *in vitro* cultivation and remain stable over several self-pollinated generations.

For this study, we used a standard MSAP analysis followed by deep amplicon sequencing using NGS technology. This combination MSAP-NGS technique not only provides information about the similarity of DNA methylation landscapes between specimens, but it also allows the extraction of sequences and the identification of genome regions that are differentially methylated between specimens. This method is relatively easy to complete and widely applicable; thus, this novel approach is rather unique compared with the currently available techniques in the epigenetics field.

## Material and Methods

### Source plant material

The common semi-dwarfing wheat cultivar Yumai66 (YM66), a 1B/1R translocation line that carries *Rht*-*B1b* and *Rht8* dwarfing genes according to previous reports [38,39], was kindly provided by the Wheat Quality Breeding Group at the Institute of Crop Sciences of Chinese Academy of Agricultural Sciences (CAAS). Seeds were sowed in the autumn of 2005 at the Experimental Station of CAAS. About 50 spikes from 50 plants was used to collect the immature embryos and cultured *in vitro* for plant regeneration in the spring of 2006 in the *in vitro* laboratory of the Institute of Crop Sciences of Chinese Academy of Agricultural Sciences in Beijing, China. Each regenerated plant was harvested and controlled for variation separately. Selected plants with promising properties were subsequently planted until a stable line was developed. The conditions for establishing the *in vitro* embryo culture were previously described in detail [40]. By this manner dwarfing somatic variation line was established and subsequently assigned as AS34. Additional crossing with other cultivars to control its potential to be used for wheat breeding was also performed [37]. Previous work also suggested that the dwarfing trait of AS34 is controlled by multiple genes, and as a parent, AS34 had positive genetic effects on main agronomic characteristics in the segregated population, including plant height [37]. The current study was performed using seeds originating from the fifth self-pollination of the initial somaclonal mutant.

At the same laboratory (Institute of Crop Sciences, Chinese Academy of Agricultural Sciences, China) and in the same manner the somaclonal variant line AS208 was established from the Chinese popular cultivar Lunxuan987 (LX987). This cultivar belongs to the group of winter wheat cultivars, has a height of approximately 80 centimeters, a close architecture, good winter hardness and high ratio of productive tillers. AS208 was very similar to its parental cultivar LX987 in its main agronomical traits; however, AS208 was missing two HMW-GSs, 1Bx20 and 1By20, encoded by the *Glu-B1* locus [36]. Therefore, AS208 is subsequently referenced as the “bread quality” somaclone. For the study material, AS208 seeds originating from the fifth self-pollination of the initial somaclonal mutant, the same generation as for AS34, were used.

### DNA isolation

Seeds from all four of the wheat genotypes (Yumai66 and its somaclonal line AS34 and Lunxuan987 and its somaclonal line AS208) were sown together in flowerpots filled with standard brown soil and periodically irrigated. Subsequently, four young plantlets of each genotype were used as a tissue source for DNA isolation. DNA was isolated using a DNeasy Plant Mini Kit (Qiagen) according to the manufacturer’s instructions. The DNA concentration was fluorescently measured using a Quant-iT Pico-Green dsDNA Assay Kit (Invitrogen). Two samples, each containing 250 ng of DNA, were prepared as input samples for subsequent digestions using the MSAP protocol (see below). Samples representing the Yumai66 standard cultivar were labeled as YM66-C1, YM66-C2, YM66-C3 and YM66-C4. Samples representing the dwarfing somaclone derived from Yumai66 were labeled as AS34-1, AS34-2, AS34-3 and AS34-4. Samples representing the Lunxuan987 cultivar were labeled as LX987-C1, LX987-C2, LX987-C3 and LX987-C4. Samples representing the “bread quality” somaclone derived from Lunxuan987 were labeled as AS208-1, AS208-2, AS208-3 and AS208-4.

### MSAP analysis

The isoschizomer pair *Hpa*II and *Msp*I was used, which recognizes tetranucleotide CCGG with differential sensitivity to methylation at the inner and outer cytosine. The first sample containing 250 ng of DNA was digested by *Eco*RI and *Msp*I restriction enzymes, and the second equivalent sample was digested by *Eco*RI and *Hpa*II restriction enzymes. Subsequent adaptor ligation and pre-amplification reactions using adaptors and primers designed for *Eco*RI and *Hpa*II/*Msp*I combinations were performed as previously described [41]. For selective amplification, three differently labelled *EcoRI*-derived primers, *EcoRI*-AGG (FAM), *EcoRI*-AGC (NED) and *EcoRI*-ACT (JOE), were combined with three *HpaII/MspI*-derived primers, *HpaII/MspI*-TCAA, *HpaII/MspI*-TCGC and *HpaII/MspI*-GCAT. Thus, a total of nine primer combinations were used. Labels were assigned for subsequent use: *HpaII/MspI*-GCAT + *EcoRI*-AGG (FAM) = combination I; *HpaII/MspI*-GCAT + *EcoRI*-ACT (JOE) = combination II; *HpaII/MspI*-GCAT + *EcoRI*-AGC (NED) = combination III; *HpaII/MspI*-TCGC + *EcoRI*-AGG (FAM) = combination IV; *HpaII/MspI*-TCGC + *EcoRI*-ACT (JOE) = combination V; *HpaII/MspI*-TCGC + *EcoRI*-AGC (NED) = combination VI; *HpaII/MspI*-TCAA + *EcoRI*-AGG (FAM) = combination VII; *HpaII/MspI*-TCAA + *EcoRI*-ACT (JOE) = combination VIII; and *HpaII/MspI*-TCAA + *EcoRI*-AGC (NED) = combination IX.

Amplified products were electrophoretically separated using the capillary system of a ABI PRISM 310 genetic analyzer (Applied Biosystems). GeneScan 500 ROX (Applied Biosystems) was used as a size standard, and POP 4 polymer (Applied Biosystems) was used as a medium for fragment separation.

### Evaluation of DNA methylation landscape based on MSAP results

An accurate interpretation of the spectra was ensured by the detailed and independent manual evaluations completed by two individuals. Moreover, each peak and its intensity were evaluated in the context of the whole group using GeneScan software (Applied Biosystems) to overlap the signals from all samples. The distribution of MSAP amplicons within individual samples (i.e., presence *vs*. absence of a given DNA fragment) was translated into a presence/absence data matrix and typed into a computer file as a binary matrix (see S1 Table). Subsequently, MSAP data originating from the digestion with *Msp*I and *Hpa*II were combined for each variant and used as a basis for the calculation of their mutual epigenetic similarity with the Nei and Li/Dice algorithm [42]. Genetic similarity/dissimilarity coefficients were computed using the UPGMA method, and the corresponding dendrograms were generated using NTSYS software (http://www.exetersoftware.com).

### Sample preparation for deep amplicon sequencing

First, we evaluated the potential of individual primer combinations to generate repeated polymorphic amplicons between the cultivars and their somaclonal lines. Based on this criterion, six primer combinations were selected for subsequent analysis (see Table 1). The reasons why only six combinations were selected are discussed below.

**Table 1:** Ability of individual primer combinations to generate polymorphic products that distinguish the original cultivar from the *in vitro* generated somaclone.

	Used isoschizomer sensitive to DNA methylation / primer combination*																	
	Msp/ I	Msp/II	Msp/III	Msp/IV	Msp/V	Msp/VI	Msp/VII	Msp/VIII	Msp/IX	Hpa^a^/I	Hpa^a^/II	Hpa^a^/III	Hpa^a^/IV	Hpa^a^/V	Hpa/VI	Hpa/VII	Hpa/VIII	Hpa^a^/IX
Number of polymorphic amplicons (within one cultivar)	1+0^b^	0+1	1+0	0+1	2+0	1+0	0	1+1	0	11 +2	1+5	7+2	3+6	2+3	0+3	3+0	3+0	1+3
Number of polymorphic amplicons (within both cultivars)	0	0	0	0	0	0	0	0	0	1+1	1+0	0	1+1	0	0	0	0	0
Total	1	1	1	1	2	1	0	2	0	15	7	9	11	5	3	3	3	4

* Sequences of individual primer combinations are specified in the Material and Methods section.

a—Samples ultimately selected for use in subsequent deep amplicon sequencing.

b–For each summation in the table, the first number represents the number of amplicons newly appearing in somaclones, and the second number represents the number of amplicon that disappeared in the somaclones when compared with the standard cultivar.

Next, 5 μl of the MSAP amplicons generated by 6 selected primer combinations were mixed together for individual wheat samples (6 x 5 = 30 μl). Subsequently, all four samples that formed a corresponding variant were blended (e.g., YM66-C1, YM66-C2, YM66-C3 and YM66-C4 samples). The mixed sample representing the standard cultivar YM66 was labeled as sample No. 3 in Table 2. Similarly prepared blends representing the AS34 dwarfing somaclone, LX987 standard cultivar and AS208 “bread quality” somaclone were labeled as sample No. 4, sample No. 1 and sample No. 2, respectively, in Table 2.

**Table 2:** Schema of the sample preparation for deep amplicon sequencing.

Sample No.	Background of sample	Used adaptor for rapid library	MID/barcode sequence
1	Mixture of LX987 standard individuals	RL8	ACGTACTGTGT
2	Mixture of AS208 “bread quality” somaclones	RL9	ACGTAGATCGT
3	Mixture of YM66 standard individuals	RL10	ACTACGTCTCT
4	Mixture of AS34 dwarfing somaclones	RL11	ACTATACGAGT

### Deep amplicon sequencing

The amplicon library was prepared according to the “Rapid Library Preparation Manual” (available on: http://454.com/downloads/my454/documentation/gs-junior/method-manuals/GSJuniorRapidLibraryPrepMethodManual_March2012.pdf) using multiplex identifiers (MIDs). The library was added to the emulsion PCR at a ratio of 0.2 molecules per bead, and the emulsion PCR amplification was performed as described in the “emPCR Amplification Method Manual–Lib-L” (available on http://454.com/downloads/my454/documentation/gs-junior/method-manuals/GSJunioremPCRAmplificationMethodManualLib-L_March2012.pdf). After a bead recovery and enrichment procedure, a picotiter plate was prepared. Approximately 500,000 enriched beads were loaded and sequenced according to the “Sequencing Method Manual” (http://454.com/downloads/my454/documentation/gs-junior/method-manuals/GSJuniorSequencingManual_Jan2013.pdf). Sequencing reactions were performed using a Roche 454 Junior Sequencer with GS Titanium Sequencing Kit according to the manufacturer's instructions (Roche Life Science).

Imaging and signal processing was competed using the GS Junior gsRunProcessor v3.0 full Processing, shotgun library pipeline.

### Methodology for extracting stable and repeated polymorphic regions from sequencing data

**Fig 1:**
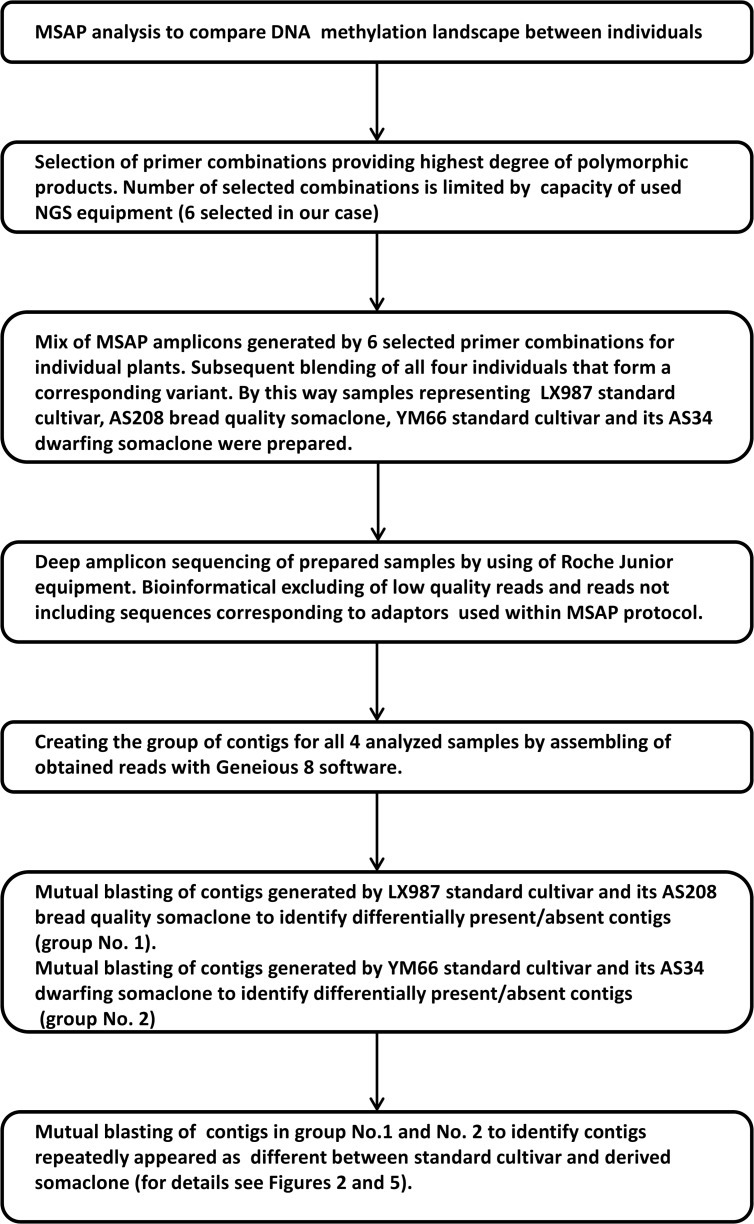
Whole experimental pipeline of newly introduced approach combining MSAP and NGS techniques

**Fig 2:**
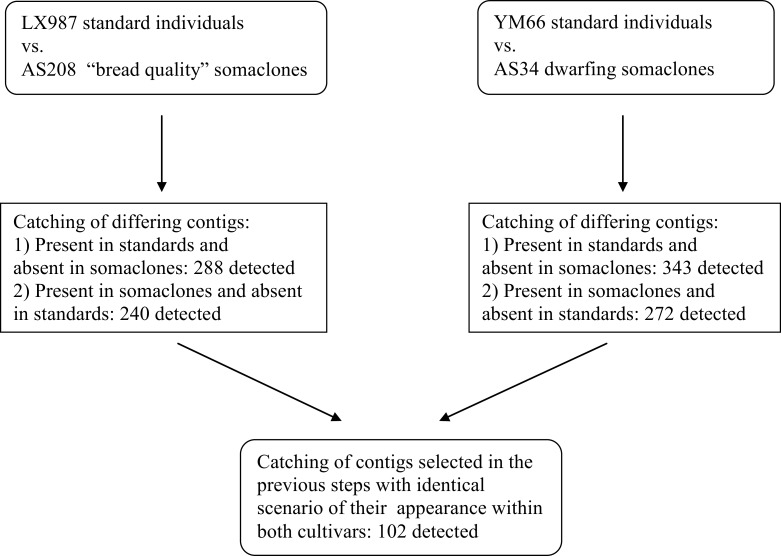
Pipeline for management of contigs to identify repeated polymorphic amplicons across the standard cultivars and their derived somaclones.

Sequence quality was verified by UNIX-based package fastQC-0.10.1 software. GS Rune Browser 3.0 software (Roche Life Science) was used for adaptor trimming, assembly of reads to contigs was performed by using Geneious 8 software (www.geneious.com). To enhance the credibility of the results the contigs with a number of reads less than 2 were excluded from further analysis. Subsequently, contigs obtained within the four samples described in Table 2 were blasted (e-value e^-5^) in accordance with previous reports [43,44]. The LX987 standard cultivar mixture (sample No. 1 in Table 2) was compared against the AS208 “bread quality” somaclone mixture (sample No.2 in Table 2) and vice versa. Similarly, the YM66 standard cultivar mixture was blasted against the AS34 dwarfing somaclone mixture and vice versa. The aim of this approach was to identify contigs that differed between the standard cultivar and its somaclone. Thus, the group of contigs that differed between LX987 and its somaclone and the group of contigs that differed between YM66 and its somaclone were established. These two groups of differing contigs were mutually blasted to identify consensual contigs. This analysis aimed to isolate contigs with differences between both sets of standard cultivars and their somaclones. Indeed, this method of analyzing high-throughput sequencing data detected contigs representing regions that were repeatedly changed by *in vitro* cultivation and remained meiotically stable. The resulting group of 102 contigs obtained by this procedure was blasted using a BLASTN search against the GenBank/NCBI. The overall experimental pipeline and approach used for extraction of the final set of blasted sequences are summarized in Figs 1 and 2.

## Results and Discussion

### Comparison of DNA methylation landscapes using MSAP spectra

The present study utilized two varieties of wheat and their somaclones with interesting and meiotically transmittable phenotypes generated under *in vitro* conditions. Some reports have described that culturing under *in vitro* conditions generated changes at the genomic level [45,46,47]; however, the direct comparison of somaclones and original cultivars frequently showed DNA methylation changes over changes in primary DNA structure [16,48,49,50]. Moreover, considerable DNA methylation changes after *in vitro* cultivation have been frequently described [15,23,51]. Thus, the somaclones investigated in this study were likely to show a significant change in their DNA methylation profile when compared with the original cultivars. Furthermore, this phenomenon may ultimately affect the expression of genes that control specific phenotypes. Originally, DNA methylation was mainly considered as part of a transient and reversible gene regulation. However, recent studies have described many examples of stress-induced epigenetic changes that are meiotically heritable [34,35].

**Fig 3:**
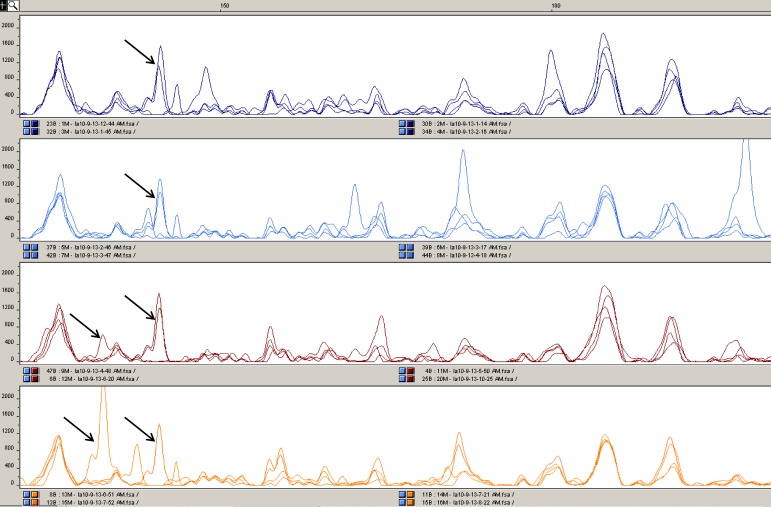
Example of MSAP spectra showing polymorphism between standard cultivar and derived somaclonal individuals. Top window shows MSAP spectra obtained by analysis of four different individuals of LX987 standard cultivar by using *HpaII/MspI*-GCAT + *EcoRI*-AGG (FAM) adaptors. Second window shows MSAP spectra obtained by analysis of four different individuals of AS208 “bread quality” somaclones by using identical adaptors as for the samples in top window. Third window shows MSAP spectra obtained by analysis of four different individuals of YM66 standard cultivar by using identical adaptors as for the samples in top window. Fourth window shows MSAP spectra obtained by analysis of four different individuals of AS34 dwarfing somaclones by using identical adaptors as for the samples in top window.

To compare the general DNA methylation landscapes of our samples, a standard MSAP analysis was first performed. Sixteen genotypes were analyzed: both cultivars were represented by four standard specimens and four somaclonal specimens. A total of 2081 amplicons showed repeatability in the spectra evaluations completed by two independent analysts. Thus, our use of nine primer combinations resulted in an average of 231 amplicons per primer combination. The average similarity coefficient was 0.9974 between the standard LX-987 cultivar specimens and 0.9986 between the derived somaclonal specimens. Similarly, the average similarity coefficient was 0.9972 between the standard YM66 cultivar specimens and 0.9982 between the derived somaclonal specimens. Consequently, the overall average similarity coefficient within each variant was 0.9979. Such high values are substantially at the level of the natural error of used MSAP method, resembling values of similarity that would be probably obtained when analyzing technical replicates of identical samples. It also means that the DNA methylation landscapes of specimens from the same variant are highly similar. A contrasting result was obtained for the comparisons between the original cultivar and its somaclone. The average coefficient of similarity between all LX-987 vs. AS208 somaclonal specimens was 0.9911. The average coefficient between all YM66 vs. AS34 specimens was 0.9853. Determination of the similarity coefficients using the algorithm for similarity calculations (Nei and Li, 1979) provided the ratio of monomorphic to polymorphic amplicons between individual variants. If we take this into account, the average percentage of polymorphic amplicons between specimens from the same variant was 0.43%. In contrast, the average percentage of polymorphic amplicons between somaclone and the original cultivar (i.e., YM66 vs. AS34 and LX-987 vs. AS208) was 2.33%. Example of MSAP spectra being polymorphic between standard cultivar and derived somaclone are presented within Fig 3.

**Fig 4:**
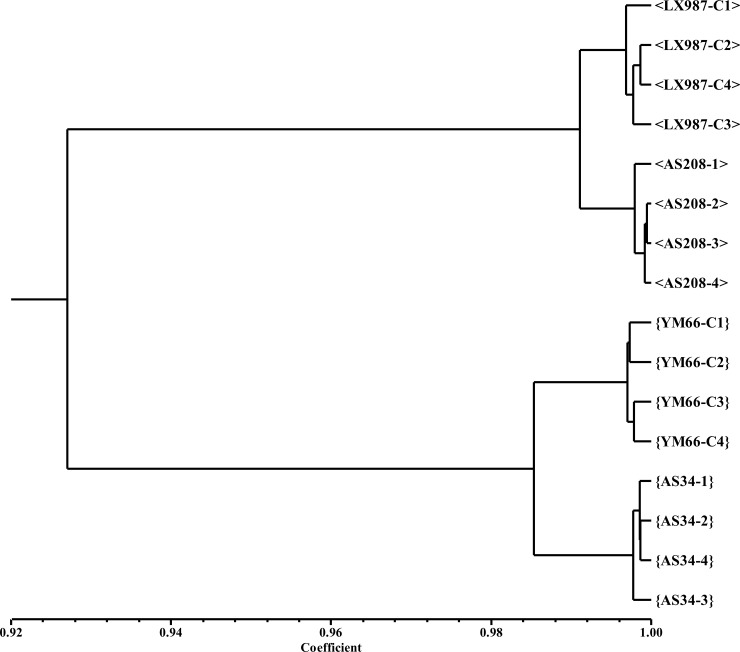
Dendrogram showing similarity of DNA methylation landscapes between samples

The similarity of DNA methylation landscapes established within the analyzed specimens using the MSAP method is presented as a dendrogram in Fig 4. The dendrogram shows a clear segregation of specimens into four distinct clusters. The first level of clustering is determined by the cultivar. Subsequent order for clustering shows that the somaclones are strictly separated from their respective standard cultivar. The dendrogram confirms the difference in DNA methylation state between specimens originating from *in vitro* cultivation and specimens from the standard variety. Unfortunately, approaches suitable for providing additional details on the character of these differentially DNA methylated regions are generally difficult to complete. One possible solution is the use of NGS techniques. Our novel approach that combined MSAP and NGS techniques and the subsequent results are described later in the text.

### Sample selection and preparation for deep amplicon sequencing

The Roche 454 platform was used for amplicon sequencing. In terms of above-average read lengths, Roche 454 is particularly advantageous for amplicon sequencing when compared with other available platforms. On the other hand, the drawback of Roche 454 is a relatively low sequencing capacity per run [52]. This disadvantage was carefully taken into account while designing our NGS experiment. Overloading the capacity of our NGS device would create the risk of falsely identified polymorphic amplicons between samples due to an inability of the device to sequence all of the amplicons.

To the best of our knowledge, we are the first to describe the method of directly combining MSAP and NGS techniques. Because we could not base our experimental design on previous data, the final number of sequenced primer combinations was estimated based on typical MSAP profile properties and our desired coverage of sequenced amplicons. We estimated that the average length of a MSAP amplicon was 200 bp and the number of amplicons generated by one primer combination was 300; therefore, the total capacity at 1x coverage for our four samples was 240,000 nucleotides (200 x 300 x 4). Larger coverage is clearly necessary for reliable results. For example, a coverage at least 10x is recommended to identify SNPs [53]. For our current study, the goal was to detect differences in appearance of whole amplicons, not SNPs. Thus, 10x coverage was more than sufficient for our purpose. To achieve this level of coverage, a capacity of 2,400,000 nucleotides is theoretically needed for one primer combination. The use of six primer combinations provides a theoretical capacity of 14,400,000 nucleotides, which is significantly below the reported 35 Mb capacity of the Roche 454 Junior Sequencer.

Based on our calculations, finally six primer combinations were used to provide detection of differing MSAP polymorphisms between standard varieties and their somaclones (see Table 1). The primer selection included all primer combinations corresponding to the polymorphic amplicons detected between the somaclone and its standard cultivar for both varieties. This type of repeatedly polymorphic amplicon has the highest potential to represent regions that consistently change during *in vitro* cultivation and remain meiotically transferable. As shown in Table 1, a total of five of these amplicons, generated by three primer combinations, were identified from standard MSAP spectra.

Table 1 also shows that more polymorphisms were successfully detected between the specimens when DNA for the PCR template was cleaved by *Hpa*II. The sensitivity of the applied enzymes (*Msp*I and *Hpa*II) towards methylation within their CCGG recognition sequence can be used to understand this phenomenon. *Msp*I can cleave non-methylated CCGG sequences and hemi- (mC in one DNA strand only) or fully methylated CmCGG sequences but not hemi- and fully methylated mCCGG and mCmCGG sequences [54,55]. *Hpa*II only digests non-methylated CCGG sequences and hemimethylated mCCGG sequences from all the possible methylated CCGG variants [54,56]. The higher prevalence of polymorphic fragments originating from *Hpa*II digestion suggests that more CG than CNG methylation polymorphisms are present within the cultivars and their somaclones. A higher percentage of *Hpa*II-derived amplicons was also reported by Lei et al. [57] and Wang et al. [58].

### Raw deep amplicon sequencing data

The basic statistics of the NGS run showed that 80,830 reads were generated for a total of 27,757,905 nucleotides. This value is two times larger than the initial theoretical estimate (14 400 000 nucleotides). Notably, the selection of six primer combinations utilized only 77.1% of the 35 Mb capacity of the device. A control bioinformatics analysis showed that the average coverage of the 102 selected contigs (see below) was 14.73x, which is more than sufficient to assess the simple presence or absence of contigs in a given sample. For future use of this method with a similar experimental paradigm (i.e., study of two samples with desired phenotypes and two controls), the sequencing of MSAP amplicons generated by using six primer combinations will provide nearly optimal results. Theoretically, 1–2 more combinations can be added to achieve 100% utilization of the NGS device’s capacity. It will, however, automatically reduce the average sequencing coverage and increase the risk of falsely identifying polymorphic amplicons.

**Fig 5:**
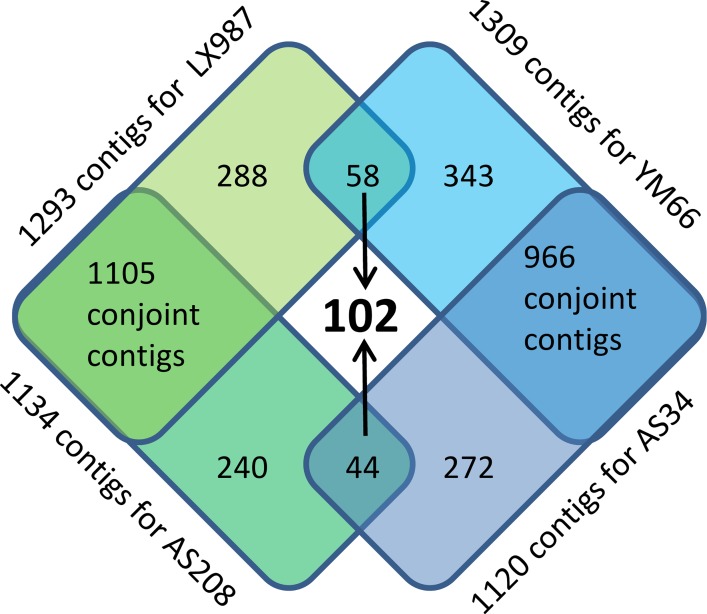
Extraction of contigs representing amplicons differentially appeared across the standard cultivars and their derived somaclones repeatedly for both pairs.

Further evaluation of the deep amplicon sequencing showed that the number of reads and contigs originating from the YM66 (sample No. 3 in Table 2) standard cultivar was 11 614/1293 respectively. For the derived dwarfing somaclone AS34 (sample No. 4 in Table 2) it was 12 038 reads and 1134 contigs. Similarly, the number of reads and contigs originating from the LX987 standard cultivar (sample No. 1 in Table 2) was 13 269/1309 respectively. For the derived AS208 quality somaclone (sample No. 2 in Table 2) it was 11 150 reads and 1120 contigs. Based on the goals of the current study, the most important contigs were those that differentially appeared between the standard cultivar and its stable somaclone. A bioinformatics analysis revealed 288 contigs that were present in the LX987 standard cultivar but absent in its somaclone. Conversely, there were 240 contigs present within the “bread quality” somaclone AS208 but absent in the standard LX987 cultivar. A total of 343 contigs were identified in the YM66 standard cultivar but not in its dwarf somaclone. Conversely, 272 contigs appeared within the dwarf somaclone AS34 but not within the YM66 standard cultivar. For a better understanding of the characteristics and molecular background of the stable epigenetic changes induced by *in vitro* cultivation, the most relevant contigs are ones that consistently showed repeated polymorphisms between the standard cultivar and its somaclone for both studied cultivars. After the exclusion of contigs that are polymorphic within one cultivar only, 102 contigs remained (see process of contig selection on Fig 5).

This number clearly reveals the potential of using MSAP in combination with subsequent NGS. Using the standard MSAP protocol alone, revealed only five polymorphic fragments in both cultivars and their somaclones. Moreover, polymorphic amplicon sequences are readily available with the combined MSAP-NGS techniques. The annotation results of the 102 contigs containing repeated polymorphisms between the standard cultivars and their somaclones are described and discussed later in the text.

### Blast of amplicons showing polymorphisms across both pairs of standard cultivars and their derived somaclones

Bread wheat (*Triticum aestivum*) is a hexaploid with three complete genomes, termed A, B and D, comprised 21 pairs of chromosomes in the nucleus of somatic cells. Each of these genomes is almost twice the size of the human genome and consists of approximately 5.5 billion base pairs. Despite recent advances in next-generation sequencing (NGS), a credible allocation of the obtained sequences to particular chromosomes in the wheat genome is lacking due to its allopolyploidy and large genome size (approximately 17 Gb). One of the successful strategies to overcome these difficulties is the interpretation of very complex genome sequencing based on complexity reduction, such as exome only capture [59,60] or genotyping by sequencing [61,62]. Another strategy utilizes flow-sorting to separate individual *Triticum aestivum* chromosomes before sequencing. This technique has enabled the development of chromosome-specific BAC libraries for the 'Chinese Spring' cultivar and is the first step in determining the whole wheat genome sequence. The IWGSC (International Wheat Genome Sequencing Consortium) is the umbrella organization managing this effort [63].

Unfortunately, the extremely complex characteristics of the wheat genome have delayed the sequencing of the entire genome and gene assignment to individual chromosomes; therefore, this information is not currently available on NCBI or similar databases. There is access to only one reference genome of chromosome 3B over a length of one gigabase [64].

The complete results for the blast search of the 102 polymorphic contigs within the NCBI database are presented in S2 Table. Some blasted contigs show similarity with relatively large contigs in the database (up to 500 kbp). Although some of these long contigs bear the name of a specific gene, they comprise many other functional regions. Therefore, we chose similarities with smaller sequences and known functions, when possible, for the final annotation of the 102 contigs. The summary of this evaluation is presented in Table 3.

**Table 3:** Characteristics of the repeatedly polymorphic contigs across somaclone and standard cultivar pairs.

Classification of repeatedly polymorphic contigs	Number of contigs found in individual classification
Identified as *T*. *aestivum* within NCBI; no functional annotation	36
Identified as *T*. *aestivum* only within Wheat Portal; no functional annotation	34
Identified as another species within NCBI; confirmed as *T*. *aestivum* within Wheat Portal;no functional anotation	8
Transposable elements	11
Chloroplast DNA	4
Individual genes; namely WPDP domain, cytokinin oxidase/dehydrogenase, lysine tRNA, pentatricopeptide containing mRNA 445 precursor, acetyl-CoA carboxylase, calmodulin, resource regulator ARR 10, MEMB 12 protein, PDI-like protein	9

The first row includes 36 contigs that show similarity with *T*. *aestivum* sequences within the NCBI database (mainly 3B chromosome); however, their functional annotation remains undescribed. Thirty-four of the 102 contigs were not assigned to any of the currently known sequences or regions within the entire NCBI database. This can be explained by the limited knowledge of the wheat genome and its annotation to functional sequences. The credibility of these 34 sequences should be emphasized because they were confirmed by subsequent blasting within another database specialized on the rapid publishing of the wheat genome sequence (The Wheat Portal administered by INRA, France; available on http://wheat-urgi.versailles.inra.fr/Seq-Repository/BLAST). All 34 sequences showed significant similarity with certain regions of wheat genome available in the database. A drawback of this database is a lack of details on chromosome annotation and the pertinent functional properties of the analyzed sequences.

Eight sequences showed no functional assignment and were identified by the NCBI database as similar to other cereal species (usually *Hordeum vulgare*). An additional blast analysis of these eight sequences was performed within The Wheat Portal database ((http://wheat-urgi.versailles.inra.fr/Seq-Repository/BLAST)). All 8 sequences were assigned to sequenced regions of the wheat genome (but again without functional evaluation). Notably, the identified sequences with currently unknown functions can be assessed in future studies. As the wheat genome is further described, the involvement of these sequences in epigenetic processes and phenotypic alterations can be established.

**Fig 6:**
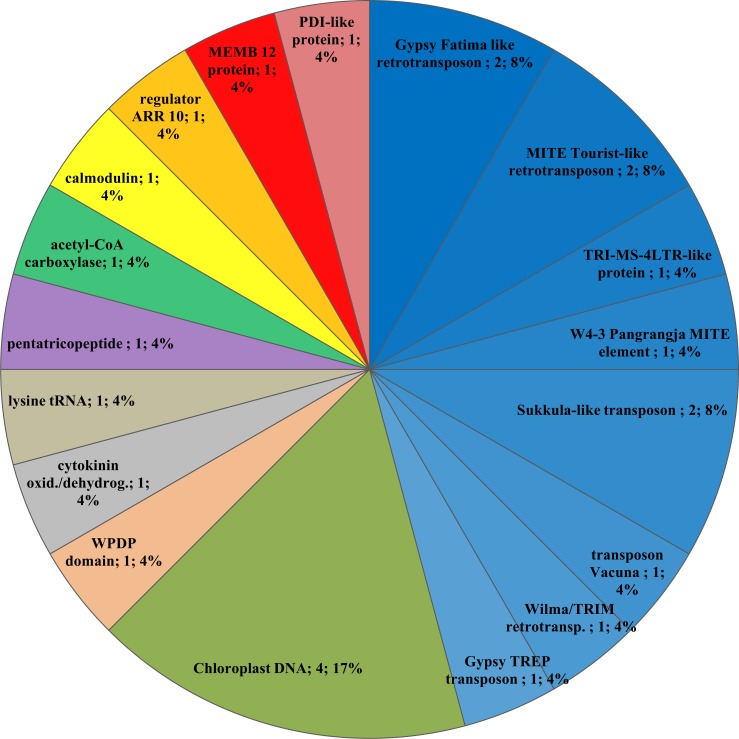
Character and frequency of repeatedly polymorphic contigs with identified role/function.

All of the remaining sequences in Table 3 showed similarity with known genes/regions (rows 4–6). Their characterization and representation is depicted in Fig 6. As it can be seen, the largest group consist of contigs containing transposable elements up to 44%. Subsequently, all of these contigs with known genes/regions were thoroughly examined in comparison with recently available literature. The aim of this comparison was to discover the theoretical role of each sequence within epigenetically driven processes in plants. No significant connections were found for 9 individual genes listed in the last row of Table 3. On the contrary, promising results were shown if potential of identified transposable elements to affect properties of somaclones was considered. Therefore mainly transposable elements will be further discussed in the subsequent text.

### Contigs representing transposable elements

Transposable elements (TEs) are ubiquitous components of almost every eukaryotic genome investigated thus far. Their representation ranges from 20% in the *A*. *thaliana* genome [65] to 85% in the maize genome [66]. Recent evidence suggests that proliferation of TEs played a role in the massive expansion of the 17 Gb bread wheat genome, with the exception of two rounds of polyploidization. The only completely sequenced wheat chromosome, 3B, consists of 85% TEs [67]. The two main types of TEs are distinguished by their transposition mechanisms: type 1 TEs (retrotransposons) transpose by an RNA intermediate, and type 2 TEs (DNA transposons) transpose by DNA. Retrotransposons are predominant in plant genomes. Both TE types consist of autonomous elements that encode all of the activities required for their movement and non-autonomous elements that transpose in response to their autonomous counterparts [68].

Newly available genomic sequence data has provided an unprecedented view on the dynamic nature of retrotransposon distribution in eukaryotic genomes and fully uncovered the crucial role of TEs in the long-term and short-term dynamics of plant genomes. Short-term dynamics are mainly associated with the activation/deactivation of transposable elements as a reaction to stressful conditions [69,70,71,72]. The strong influence of epigenetic factors, including DNA methylation, is purported to play a role in the activation/deactivation of TEs [73].

In the light of these findings, the significant presence of transposable elements in our experiment was not surprising. The contigs identified as repeatedly polymorphic between the standard cultivar and its somaclone contained multiple TEs: Gypsy Fatima like retrotransposon (sequence No. 31 and 63 in S2 Table), MITE Tourist-like retrotransposon (sequence No. 35 and 43 in S2 Table), TRI-MS-4LTR-like protein (sequence No. 42 in in S2 Table), W4-3 Pangrangja MITE element (sequence No. 44 in in S2 Table), Sukkula-like transposon (sequence No. 51 and 98 in in S2 Table), transposon Vacuna (sequence No. 76 in in S2 Table), Wilma/TRIM M20B retrotransposon (sequence No. 81 in in S2 Table), and Gypsy TREP 3173 Derami transposon (sequence No. 88 in in S2 Table). To obtain more comprehensive information, reverse search aimed to extract contigs containing transposable elements repeatedly appearing within all analyzed samples was also performed, resulting in identification of 7 such contigs. Specifically, they were CACTA transposon (1x); gypsy-like LTR-retrotransposon Ashbury-2 (1 x); MITE-like transposon Damocles_N11D-2 (1x); LINE-like transposable lement (1x); retrotransposon Gypsy TREP 3196_Fatima (3x).

Stable somaclones that changed phenotypes were used in this study; thus, it is important to note that the transposing of TEs can significantly alter important plant characteristics if genes or regulatory regions are affected. In one of the most famous examples in the plant kingdom, berry color was changed by transposition in a pathway for the synthesis of anthocyanins in vines [74]. An economically important example within somaclonal variants arising from tissue cultures consisted of a “mantled” oil palm fruit abnormality that drastically reduced yield and thus has largely halted efforts to clone elite hybrids for oil production. Recently, studies showed that DNA hypomethylation of a LINE retrotransposon related to rice *Karma*, in the intron of the homeotic gene *DEFICIENS*, significantly contributed to the induction of this “mantled” phenotype [75]. With regards to the dwarfing phenotype observed in our study, sequence No. 44 in S2 Table appears to be the most relevant. This sequence shows similarity with the Pangrangja MITE element and part of a sequence that includes the transcription factor of the Lks2 gene, which drives the phenotype of short internodes in barley awns [76].

## Conclusion

The interlacing of epigenetically induced changes remains limited, mainly due to the availability of suitable and effective techniques. The novel approach of combined MSAP and NGS techniques allows for the simultaneous generation of basic information on the similarity of DNA methylation landscapes between genotypes and identification of polymorphic DNA sequences. We see the highest potential of this approach in studies consisting of closely related specimens (clones, somaclones) with a contrasting phenotype. In this type of study, the number of identified polymorphic amplicons will likely be manageable and facilitate the development of new and promising topics or directions for subsequent research.

In our study, we identified 102 amplicons that were polymorphic across stabilized somaclones and their standard cultivars. Unfortunately, an important portion of these identified amplicons have no functional annotation because of the very complex hexaploid character of the wheat genome. However, the functional roles of these sequences can be determined in future studies, as the wheat genome continues to be sequenced. Based on the DNA methylation landscapes, transposable elements were identified as the most significant factors involved with changing somaclone properties.

## Supporting Information

S1 Table MSAP evaluation. (XLSX)

S2 Table Results of blasting. (XLSX)
